# Minimizing unnecessary brain magnetic resonance imaging in pediatric endocrinology: a retrospective cohort analysis

**DOI:** 10.3389/fendo.2024.1456541

**Published:** 2024-09-03

**Authors:** Maura Marin, Flora Maria Murru, Francesco Baldo, Gianluca Tamaro, Elena Faleschini, Egidio Barbi, Gianluca Tornese

**Affiliations:** ^1^ Department of Medicine, Surgery and Health Sciences, University of Trieste, Trieste, Italy; ^2^ Institute for Maternal and Child Health IRCCS “Burlo Garofolo”, Trieste, Italy

**Keywords:** brain magnetic resonance imaging, incidentaloma, growth hormone deficiency, central precocious puberty, follow-up

## Abstract

**Background:**

Brain magnetic resonance imaging (MRI) is mandatory or highly recommended in many pediatric endocrinological conditions to detect causative anatomic anomalies and rule out neoplastic lesions. However, MRI can also show findings associated with the underlying clinical condition, as well as unrelated “incidentalomas”. These latter findings are often abnormalities with a high incidence in the general population for which there is no clear literature regarding their management, especially in pediatric patients. The present study aimed to evaluate the number of unnecessary performed MRIs in pediatric endocrinology.

**Methods:**

Retrospective analysis on 584 MRI scans performed in 414 patients (254 growth hormone deficiency, 41 other causes of short stature, 116 central precocious puberty).

**Results:**

The MRI scans were completely normal in 67% of the individuals, and the prevalence of individuals who underwent more than one MRI was 18%, with no significant differences among the groups. The overall prevalence of incidentalomas was 17%. Among 170 repeated MRI scans, 147 (86%) were not required according to a dedicated protocol. Only five patients (four GHD, one Noonan) correctly repeated the MRI. All the repeated MRI scans did not reveal any progression in the findings. If we include the MRIs performed in cases of OCSS other than Noonan syndrome (n=32) and girls with CPP older than 6 years (n=89), an additional 121 MRIs could have been avoided, leading to a total number of unnecessary MRIs to 268 (46%).

**Conclusions:**

Only a few specific neuroimaging findings in endocrinologic pediatric patients warrant further investigation, while too often repeated imaging is carried out unnecessarily. We advocate the importance of guidelines to reduce costs for both the healthcare system and patients’ families, as well as to alleviate physical and psychological distress for patients and caregivers.

## Introduction

Brain magnetic resonance imaging (MRI) is mandatory or highly recommended to detect causative anatomic anomalies and rule out neoplastic lesions in many pediatric endocrinological conditions, such as central precocious puberty (CPP) (1) and growth hormone deficiency (GHD) (2) or other causes of short stature (OCSS) in which recombinant human growth hormone (rhGH) is prescribed such as Noonan syndrome (3). Often, previously unsuspected anomalies and malformations are found at brain MRI and sometimes these findings are completely unrelated to the clinical aspect for which the radiological examination was requested (4, 5). In the latter case, such a finding is referred to as “incidentaloma”. Due to the lack of clear pediatric guidelines for the management and follow-up of these radiological findings, sometimes the use of radiological exams might become excessive and even disproportionate to the patient’s medical needs. In a previous study, we reported the suggested management of the most frequent brain findings found in pediatric patients affected by GHD and CPP (6), which would result in a reduction of not only economic but also physical and psychological implications. The present study aimed to evaluate the number of unnecessary performed MRIs in pediatric endocrinology. Our goal was to highlight that, based on the current literature, most of the follow-up brain MRIs are probably not required and that only a few neuroimaging findings are worth subsequent investigations.

## Materials and methods

This is a retrospective cohort study on children who had a brain MRI with pituitary protocol performed at the Institute for Maternal and Child Health IRCSS “Burlo Garofolo” in Trieste, Italy, from 01/07/2007 to 31/12/2020, because of a diagnosis of CPP, GHD, or OCSS conditions in which rhGH is prescribed (i.e., Turner syndrome, chronic renal insufficiency, Prader–Willi syndrome, SHOX deficiency, children born small for gestational age [SGA] without catch-up growth, Noonan syndrome, and idiopathic short stature [ISS] cleared by the regional GH commission) (7). GHD was diagnosed in children with growth defects (height ≤−3 SDS; or height ≤−2 SDS and growth velocity ≤−1 SDS; or growth velocity ≤−2 SDS or ≤−1.5 SDS after 2 consecutive years, even without short stature) based on positive arginine and insulin stimulation tests; a GH peak below 8 μg/L in both tests was considered as pathological (8); sex steroid priming was not used since it is not recommended in our national guidelines; and patients with already known multiple pituitary deficits were excluded from the study. PPC was diagnosed in patients with secondary sexual characteristics before 8 years of age in girls and 9 years in boys with a GnRH test showing an LH peak >5 mU/mL and/or an LH/FSH peak ratio >1. In accordance with our internal protocol, all OCSS patients received an MRI scan prior to commencing rhGH therapy to rule out any preexisting brain tumors.

Patients with previously known abnormal brain MRI scans requiring follow-up (n=8) were excluded from the study. The “G2 clinico” platform (management system specialist activities) was employed to access all patients’ data. Information retrieved included date of birth, sex, indication for brain MRI (GHD, OCSS, CPP), date of brain MRI, and found abnormalities (if any). If multiple MRIs were performed, the number of MRIs, any changes in the findings, and the date of the last MRI were recorded.

Brain MRI with gadolinium-based contrast agents and with specific sequences for the hypothalamic–pituitary region was performed with a 1.5 Tesla Ingenia MR scanner (Philips Healthcare, Best, The Netherlands). Imaging in static mode with axial T2-, T1- and FLAIR (fluid-attenuated inversion recovery)-weighted sequences of the whole brain and subsequent thin-layer evaluation targeted to the pituitary gland in the sagittal and coronal planes, with T2- and T1-weighted sequences, were performed before and after administration of a full-dose contrast agent. Coronal scans allowed visualization of the pituitary gland, pedicle, chiasm, and parasellar regions whereas sagittal images were more suitable for evaluation of the midline plane. The acquisition procedure lasted an average of 20 min–30 min, and sedation was considered as a protocol in children <8 years of age (between 7 and 8 years was evaluated on a case-by-case basis). All the MRI scans were reviewed by an expert pediatric radiologist (FMM) with more than 15 years of experience.

The guidelines for the management and follow-up of MRI brain findings were first introduced in January 2021 (6). The following findings were considered as “alterations with definite or possible clinical/anatomical significance” (ADPCAS): adenohypophysis hypoplasia, pituitary stalk interruption syndrome (PSIS), ectopic neurohypophysis, complete or partial empty sella; Rathke cleft cyst (RCC), pituitary adenoma, craniopharyngiomas (or other tumors in the hypothalamus–pituitary region), and Arnold-Chiari type I. The following findings were included within the group “alterations without clinical significance/incidentalomas”: arachnoid cyst, pineal cyst, choroid plexus cysts, vascular abnormalities, and increased pituitary volume. Other findings with low frequency (n ≤ 4) and not related to GHD and CPP were grouped as “other”. The following findings were considered as “alterations worthy of radiological follow-up”: (1) craniopharyngioma or other tumors (follow-up after surgery ± radio/chemotherapy according to the oncological guidelines); (2) pituitary adenoma: if symptomatic, surgical or medical therapy + MRI follow-up according to the oncological guidelines; if asymptomatic and ≥10 mm, MRI once per year for 3 years and then every 1–2 years, if asymptomatic and ≥5 mm (<10 mm), a single MRI after 1 year; (3) RCC: if symptomatic, radiologic follow-up for at least 5 years postsurgery; if asymptomatic and >5 mm, MRI at 1, 3, and 5 years (regardless of characteristics); (4) arachnoid cyst, if large and in high-risk regions; (5) pineal cysts, if >14 mm and/or with an abnormal radiological pattern or clinical symptoms.

Ethical Committee approval was not requested since General Authorization to Process Personal Data for Scientific Research Purposes (Authorization no. 9/2014) declared that retrospective archive studies that use ID codes, preventing the data from being traced back directly to the data subject, do not need ethics approval (9). Informed consent was signed by parents at the first visit, in which they agreed that “clinical data may be used for clinical research purposes, epidemiology, the study of pathologies and training, to improve knowledge, care and prevention”. All data were collected in an anonymous database.

All statistical analyses were conducted with JMP™ (version 16.1.0, SAS Institute Inc., Cary, NC, United States). Descriptive statistics was used to describe data. Continuous variables were expressed as median with interquartile range, minimum, and maximum. Categorical data were expressed as percentages (%). For the comparison of continuous variables between more than two groups, the Kruskal–Wallis test was used. The chi-square test was used to compare categorical variables between the groups. Statistical significance was considered for p-values <0.05.

The study has been reported in line with the STROBE statement for observational studies (10).

## Result

During the study period, a total of 584 MRI scans were performed on 414 patients: 257 GHD, 41 OCSS, 116 CPP. In the OCSS group, 16 were SGA (of which 3 Silver–Russell syndrome), 9 were Noonan syndrome, 7 were ISS, 3 were Turner syndrome, 3 were SHOX deficiency, 2 were IRC, and 1 was Prader–Willi syndrome.

Clinical and imaging features of the entire cohort and the three groups are presented in **Table 1**. Age at presentation was lower in CPP and OCSS (median 8.7 and 9.1 years, respectively) compared with GHD (median 11.5 years) (p<0.01), whereas CPP had a higher prevalence of women (83%) compared with GHD (37%) and OCSS (46%) (p<0.01). In the CPP group, 7 out of 96 women (7%) were diagnosed below the age of 6 years.

**Table 1:** Clinical and imaging features of the entire cohort and the three groups (* and in bold: alterations worthy of radiological follow-up.

	Total (n=414)	GHD (n=257)	OCSS (n=41)	CPP (n=116)	p
Age at presentation (years) (median, IQR)	9.7 (7.9;12.5)	11.5 (8.2;13.2)	9.1 (5.9;11.7)	8.7 (8.0;9.3)	**<0.01**
Female (n, %)	210 (51%)	95 (37%)	19 (46%)	96 (83%)	**<0.01**
***Completely normal MRI***	276 (67%)	167 (65%)	28 (68%)	81 (70%)	0.63
***Any finding at MRI***	138 (33%)	90 (35%)	13 (32%)	35 (30%)	
Alterations with definite or possible clinical/anatomical significance (ADPCAS)					
Overall	66 (16%)	52 (20%)	3 (7%)	11 (9%)	**<0.01**
• Arnold-Chiari type 1	17 (4%)	12 (5%)	–	5 (4%)	0.46
- <7 mm	14 (3%)	9	–	5	
- ≥7 mm	3 (<1%)	3	–	–	
• Rathke cleft cyst (RCC)	15 (4%)	9 (4%)	2 (5%)	4 (3%)	0.90
- Asymptomatic <5 mm	13	7	2	4	
- Asymptomatic ≥5 mm*	**2**	**2**	–	–	
- Symptomatic*	–	–	–	–	
• Pituitary stalk interruption syndrome (PSIS)	13 (3%)	14 (5%)	–	–	**0.02**
• Pituitary adenoma	10 (2%)	6 (2%)	–	4 (3%)	0.65
- <5 mm	9	5	–	4	
- 5 mm–10 mm*	–	–	–	–	
- >10 mm*	**1**	**1**	–	–	
• Empty sella	8 (2%)	9 (4%)	–	–	0.08
- Partial	8	8	–	–	
- Complete	–	–	–	–	
• Adenohypophysis hypoplasia	8 (2%)	8 (3%)	–	2 (2%)	0.08
• Ectopic neurohypophysis	3 (<1%)	3 (2%)	–	–	0.39
• Tumors*	**2 (<1%)**	**1 (<1%)**	**1 (<1%)**	–	0.15
• Septo-optic dysplasia	1 (<1%)	1 (<1%)	–	–	0.73
Incidentalomas					
Overall	92 (22%)	55 (21%)	10 (24%)	27 (23%)	0.86
• Arachnoid cyst	22 (5%)	15 (6%)	2 (5%)	5 (4%)	0.82
- Small cyst	22	15	2	5	
- Large cyst or localization at risk of hydrocephalus*	–	–	–	–	
• Pineal cyst	18 (4%)	15 (6%)	–	3 (3%)	0.13
- <14 mm, without abnormal radiological pattern, without clinical symptoms	18	15	–	3	
- >14 mm and/or with an abnormal radiological pattern or clinical symptoms*	–	–	–	–	
• Vascular anomalies	11 (3%)	4 (2%)	2 (5%)	5 (4%)	0.20
- Developmental venous anomalies	11	5	3	5	
- Cavernomas and arteriovenous malformations	–	–	–	–	
• Increased hypophyseal volume	11 (3%)	6 (2%)	–	5 (4%)	0.29
• Choroid plexus cyst	8 (2%)	4 (2%)	–	4 (3%)	0.30
• Other	25 (6%)	10 (4%)	7 (17%)	8 (7%)	**<0.01**

CPP, central precocious puberty; GHD, growth hormone deficiency; MRI, magnetic resonance imaging; OCSS, other causes of short stature)

**Figure 1:**
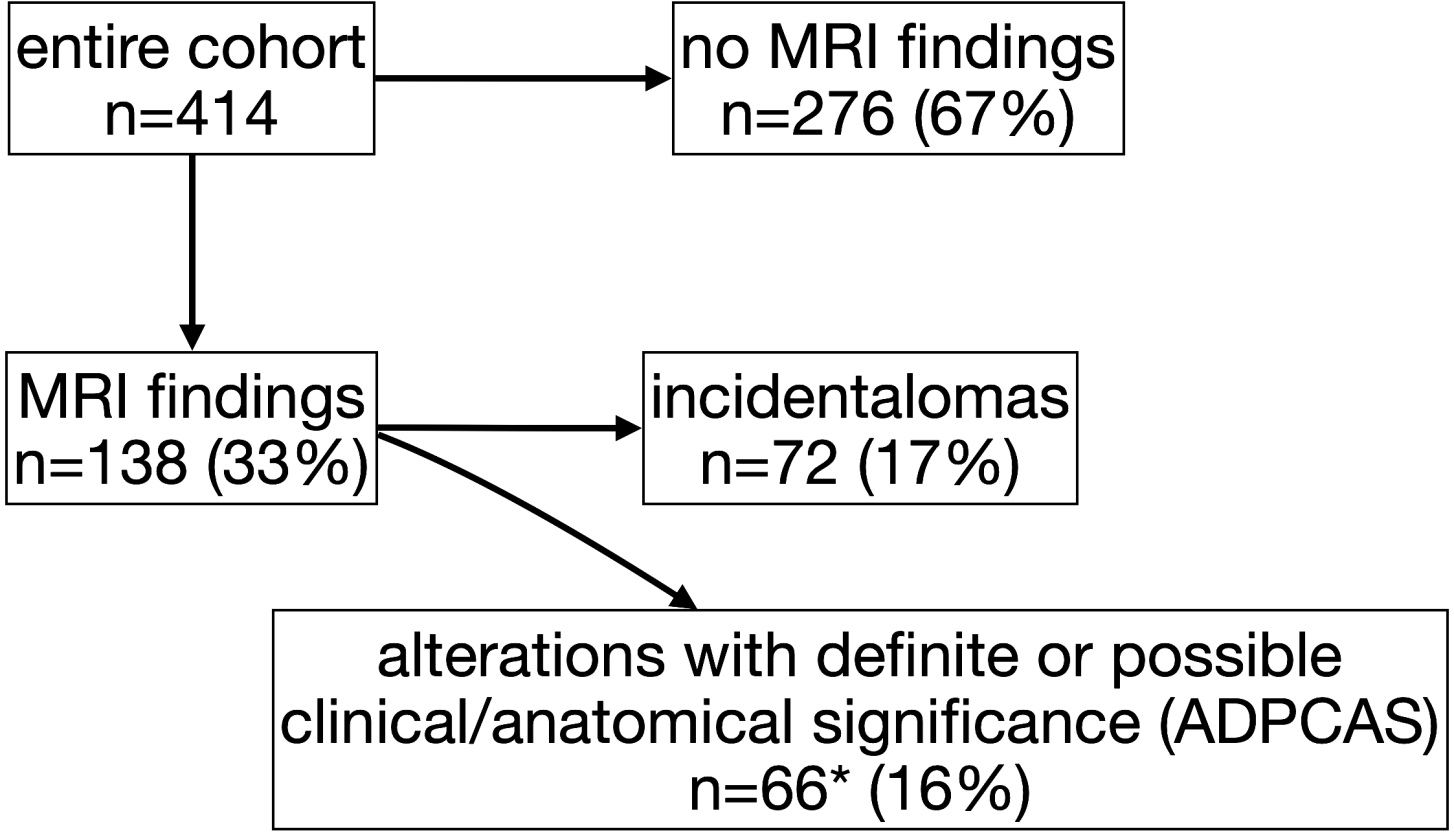
Flow diagram of study participants and magnetic resonance imaging (MRI) findings (*includes 20 patients whit also “incidentalomas”).

MRI scan was completely normal in 67% of the individuals (n=276) (**Figure 1**), with no significant differences among groups (GHD 65%, OCSS 68%, CPP 70%, p=0.63), whereas in 33% of the cases, a finding was reported (**Table 1**). ADPCAS were found in 16% (n=66) of the entire cohort (**Figure 1**): the GHD group had a higher prevalence (21%), compared with OCSS (7%) and CPP (9%) (p<0.01); in particular, PSIS (n=13), empty sella (n=8), adenohypophysis hypoplasia (n=8), and ectopic neurohypophysis (n=3) were only found in the GHD group (**Table 1**). Arnold–Chiari type 1 malformation, pituitary adenoma, and adenohypophysis hypoplasia were also detected in CPP, whereas RCC were found in both CPP and OCSS (1 Noonan, 1 ISS) patients, with no significant differences among groups (**Table 1**). No craniopharyngiomas were detected during the study period, but a dysgerminoma was found in a patient with GHD and a dysembryoplastic neuroepithelial tumor (DNET) in a patient with Noonan syndrome.

There was no significant difference in the median age between GHD patients with ADPCAS (11.3 years [6.7; 13.7]) and those without ADPCAS (11.6 years [8.57; 13.16]) (p=0.82). Similarly, the median age of CPP patients with APCAS (8.2 years [7.9; 9.2]) was not significantly different from those without APCAS (8.7 years [8.0; 9.3]) (p=0.39).

The overall prevalence of incidentalomas was 17% (**Figure 1**)—22% if we consider also those who had both incidentalomas and ADPCAS (n=92)—with no significant differences among groups (p=0.79). OCSS had a higher prevalence (21%) of “other” findings compared with CPP (7%) and GHD (6%): most of them were related to the underlying syndrome (**Table 1**).

The prevalence of individuals who performed more than one MRI was 18% (n=73), with no significant differences among diagnostic groups (GHD 19%, OCSS 10%, CPP 18%, p=0.37) (**Table 2**); the prevalence of patients with ADPCAS that performed more than one MRI (57%) was significantly higher than those without ADPCAS (10%, p<0.01). The median number of MRIs was 3, with a maximum of 11 in GHD, 7 in OCSS, and 5 in CPP, and the median distance from the first to the last MRI was 1.6 years (**Table 2**). The number of MRIs in patients with ADPCAS (median 4 [IQR 2;5], max 11) was significantly higher than in patients without ADPCAS (median 2 [IQR 2;3], max 6) (p<0.01).

**Table 2:** Data regarding repeated imaging (CPP, central precocious puberty; GHD, growth hormone deficiency; MRI, magnetic resonance imaging; OCSS, other causes of short stature) .

	Total (n=414)	GHD (n=257)	OCSS (n=41)	CPP (n=116)	p
Individuals with more than one MRI performed (n, %)	73 (18%)	48 (19%)	4 (10%)	21 (18%)	0.37
No. of MRI when >1 (median, IQR; max)	3 (2;4–max 11)	3 (2;4–max 11)	3 (2;6–max 7)	3 (2;3–max 5)	0.69
Distance from first to last MRI (years) (median, IQR)	2.0 (0.7;4.2)	2.7 (0.7;4.9)	1.9 (0.7;4.3)	1.0 (0.5;2.1)	0.11
Number of MRI with required follow-up (n, %)	5 (1%)	4 (2%	1 (2%)	–	0.33

According to our guidelines (6), only in five patients (7% of those with more than one performed MRI), four with GHD, and one with Noonan syndrome, a follow-up was required: two Rathke cleft cysts >5 mm, one non-functioning pituitary adenoma >10 mm, one dysembryoplastic neuroepithelial tumor, and one dysgerminoma. No patients with CPP or OCSS would have required a follow-up MRI. Details are reported in **Table 3**.

**Table 3:** Clinical and imaging features of lesions with indicated radiological follow-up (DNET, dysembryoplastic neuroepithelial tumor; GHD, growth hormone deficiency; RCC, Rathke’s cleft cyst) .

Patient	Sex	Diagnosis	Age at diagnosis	Finding	Number of MRIs performed
1	F	GHD	12.1 years	Dysgerminoma	9
2	M	Noonan syndrome	13.5 years	DNET	7
3	F	GHD	12.1 years	Pituitary adenoma >10 mm	4
4	F	GHD	12.1 years	RCC > 5 mm	4
5	F	GHD	11.8 years	RCC > 5 mm	3

Overall, out of 170 repeated MRI scans, 147 (86%) were unnecessary, as none of these scans showed any progression of findings. Considering an estimated cost of 450 euros for each MRI (of which 46.15 euros are paid by the patient’s families), 66,150 euros could have been saved for unnecessary tests during the study period (6,785.05 by the families and 59,365.95 by the National Health System).

If we include the MRIs performed in cases of OCSS other than Noonan syndrome (n=32) and girls with CPP older than 6 years (n=89), an additional 121 MRIs could have been avoided. This brings the total number of unnecessary MRIs to 268 (46%) and the potential savings to 130,950 euros 12,368.20 euros by families and 108,231.80 euros by the National Health System). These figures do not include the costs associated with procedural sedations in younger children.

## Discussion

Brain MRI is essential in many pediatric endocrinological conditions, such as GHD or CPP. However, it is not uncommon (13%–18%) (11, 12) to identify “incidentalomas”, incidental findings serendipitously diagnosed in a patient undergoing imaging for an unrelated reason (13). Even the majority of alterations with definite or possible clinical or anatomical relevance (ADPCAS) do not progress over time and thus would not need radiological follow-up (6). In our previous paper, we emphasized that there was a lack of guidelines regarding the management of these findings in the pediatric population, often leading to repeated MRI scans, excessive and disproportionate to the patient’s needs (6), potentially leading to significant resource expenditure and patient anxiety (14). To mitigate this risk, we have summarized the optimal management strategies for the most frequently identified alterations in patients with GHD and CPP. These strategies could not only reduce costs but also alleviate physical and psychological implications (6). Moreover, performing a brain MRI without a proper indication also raises ethical issues; written informed consent should be obtained after a detailed explanation of the reasons and the risks and benefits (15).

In this retrospective study, we evaluated the number of repeated MRIs performed and estimated the number of avoidable MRIs before the introduction of guidelines for the management and follow-up of brain MRI findings in January 2021 (6). During the study period, we identified a total of 584 MRI scans that were performed on 414 patients with GHD, CPP, or OCSS. The MRI scan was completely normal in two-thirds of the patients, and we did not observe significant differences among the three diagnostic groups (GHD, CPP, OCSS). These results confirm those obtained in many other studies on the topic, where normal MRI represents the most common outcome in children diagnosed with GHD and CPP (16–19).

Nevertheless, one-third of young individuals undergoing a brain MRI will have findings in the report that need explanation and may require follow-up, potentially causing additional anxiety for the family (14). Half of these individuals (17% of the entire cohort) had an “incidentaloma,” with no significant differences in prevalence among groups. Although the number of patients with incidentalomas who underwent follow-up MRIs was significantly lower than those with ADPCAS (10% vs. 57%), and patients with incidentalomas underwent fewer follow-up MRIs compared with those with ADPCAS (median 2 vs. 4), they all performed multiple MRI without a clear indication.

The prevalence of ADPCAS was significantly higher in children with GHD (20%) compared with those with CPP and OCSS (9% and 7%, respectively). PSIS, empty sella, ectopic neurohypophysis, and septo-optic dysplasia were observed exclusively in cases of GHD (2, 20). On the other hand, Arnold-Chiari type 1 malformation (21), pituitary adenoma (22), and adenohypophysis hypoplasia (18) were also detected in CPP patients, whereas RCCs were also found in both CPP and OCSS patients (one Noonan, one ISS), as previously reported (23, 24). No craniopharyngiomas were detected during the study period, despite GHD or CPP often being its first sign of presentation, and MRI is strongly recommended by guidelines to rule out neoplastic lesions (18–20, 25). However, we did identify a dysgerminoma in a patient with GHD and a DNET in a patient with Noonan syndrome. This latter finding represents a rare intracranial tumor, already described in patients with Noonan syndrome (26). Although the median age at GHD diagnosis might seem higher than expected (27, 28), it is consistent with other Italian reports (29, 30), suggesting a possible delay in referrals and diagnosis in Italy. Furthermore, the median age of GHD patients with ADPCAS (indicating definite GHD) was similar to that of patients without ADPCAS (who might be classified as having short stature unresponsive to stimulation tests) (31), corroborating the fact that there is no an error in diagnosis.

Overall, 147 out of 170 repeated MRI scans (86%) were unnecessary, since none of these findings would have needed a follow-up according to current literature, and none showed any progression of findings. An appropriate evidence-based follow-up was carried out in only five cases (32–37). Therefore, adherence to the criteria outlined in our study for follow-up could have reduced costs (66,150 euros) and psychological stress for the patients that imaging studies inevitably entail. According to the guidelines we proposed in our previous study (6), brain MRI findings that did not require radiological follow-up could be adequately managed with clinical and laboratory monitoring alone. Among ADPCAS, the management of an empty sella warrants further discussion, as there is a significant gap in evidence-based guidelines. Some authors advocate for radiological follow-up due to the theoretical risk of progression (38, 39). However, given the very low incidence of neuroradiological progression, which correlates with hormonal deterioration, clinical and laboratory monitoring alone may be sufficient for these patients (39). If new signs, symptoms, or hormonal changes suggestive of progression occur, an MRI evaluation becomes necessary. Our data have revealed that all findings that underwent more than one MRI remained stable over time, suggesting, therefore, that extensive follow-up is not necessary when they do not initially present insidious characteristics.

In addition to reconsidering repeated MRIs, it is important to question the necessity of performing an MRI initially. While the indication for neuroimaging in GHD is clear—to identify anatomical anomalies that may explain the etiology and to exclude neoplastic lesions that could contraindicate rhGH therapy (2)—there are still controversies about the necessity of performing MRI scans on all children with CPP. The likelihood of finding significant intracranial abnormalities in girls over 6 years old is low, although not zero (1, 40). In our cohort, we did not find any neoplastic lesions in CPP patients. By excluding girls over the age of six from MRI screenings, we could have avoided 89 tests.

It is not uncommon to perform an MRI before starting treatment with rhGH in non-GHD patients to rule out neoplastic lesions (3, 41, 42), even though no published guidelines support the routine use of brain MRI in these cases. It should be noted that in conditions where rhGH is indicated, such as Turner syndrome, Noonan syndrome, or *SHOX* deficiency, GHD is typically not excluded, although coincidences have been reported (43–45). Furthermore, the rate of abnormal MRI findings is similar in short children with normal GH responses and normal IGF-1 levels compared with children with GHD (46). Currently, evidence does not support the use of MRI of the pituitary region in short children born SGA without GHD (47), whereas MRI is recommended in children with Noonan syndrome (3), due to their higher risk of developing tumors, including brain tumors, compared with the general population (48, 49). In our cohort, a DNET was found in a patient with Noonan syndrome. Therefore, we recommend a precautionary brain MRI before initiating rhGH therapy in NS patients to exclude any preexisting brain tumors. In other patients with OCSS, an MRI might not be necessary, unless they present neurological symptoms or signs of hypopituitarism. With this approach, we could have spared an additional 32 MRIs.

Overall, we could have spared 268 MRIs (46% of the total number), resulting in potential savings of more than 130,000 euros, not including the costs for procedural sedations. By fine-tuning our internal protocol, we aim to further reduce the number of MRIs, including repeat scans, in the coming years.

While our study offers valuable insights, it is not without limitations. Primarily, it relies on retrospective data. However, we followed meticulous steps to mitigate potential biases. All available data were gathered to minimize selection bias, and to ensure objectivity, every MRI was scrutinized by an expert pediatric radiologist, mitigating reporting bias. Additionally, being a single-center study, there is a possibility that our findings may not fully represent broader populations. Nevertheless, the consistency of our results with prior literature suggests potential generalizability. Lastly, a notable constraint arises from the absence of a “healthy” control group among the pediatric population undergoing neuroimaging solely for pituitary disorders. This limitation is inherent in many similar studies in the literature exploring brain alterations in children with pituitary-related diseases (4). On the other hand, to our knowledge, this is the first study evaluating the follow-up of MRI findings in children with endocrine disorders. There is a lack of guidelines on incidentalomas and MRI brain abnormalities in the pediatric population. Therefore, our study can support many pediatricians who daily find themselves having to manage such clinical conditions.

With this study, we confirm that only a few neuroimaging findings in pediatric patients warrant further investigation, and we highlight that too often investigations are carried out unnecessarily. We advocate the importance of being familiar with guidelines for prescribing these exams and managing these findings to reduce costs for both the healthcare system and patients’ families, as well as to alleviate physical and psychological distress for patients and caregivers.

## Data Availability

The raw data supporting the conclusions of this article will be made available by the authors, without undue reservation.
